# *N*-{(2*S*)-3-Hy­droxy-4-[(5-methyl-1,3,4-thia­diazol-2-yl)sulfan­yl]-1-phenyl-2-but­yl}-4-methyl­benzene­sulfonamide

**DOI:** 10.1107/S1600536811033575

**Published:** 2011-08-27

**Authors:** Claudia R. B. Gomes, Thatyana R. A. Vasconcelos, Walcimar T. Vellasco Junior, Wilson Cunico, James L. Wardell, Solange M. S. V. Wardell, Edward R. T. Tiekink

**Affiliations:** a Instituto de Tecnologia em Fármacos - Farmanguinhos, FioCruz - Fundação, Oswaldo Cruz, R. Sizenando Nabuco, 100, Manguinhos, 21041-250, Rio de Janeiro, RJ, Brazil; b Universidade Federal Fluminense, Instituto de Química, Departamento de Química Orgânica, Outeiro de São João Batista, s/no, Centro, Niterói, 24020-141, Rio de Janeiro, Brazil; c NuQuiA - Núcleo de Qímica Aplicada, Departamento de Química Orgânica, UFPel, Campus Universitário s/n, 96010-900 Pelotas, RS, Brazil; d Centro de Desenvolvimento Tecnológico em Saúde (CDTS), Fundação Oswaldo Cruz (FIOCRUZ), Casa Amarela, Campus de Manguinhos, Av. Brasil 4365, 21040-900, Rio de Janeiro, RJ, Brazil; e CHEMSOL, 1 Harcourt Road, Aberdeen AB15 5NY, Scotland; f Department of Chemistry, University of Malaya, 50603 Kuala Lumpur, Malaysia

## Abstract

The thia­diazoyl and sulfonyl-benzene rings in the title compound, C_20_H_23_N_3_O_3_S_3_, are aligned to the same side of the mol­ecule, forming a twisted ‘U’ shape [dihedral angle = 77.6 (5)°]. The benzyl-benzene ring is orientated in the opposite direction from the mol­ecule but projects approximately along the same axis as the other rings [dihedral angle between benzene rings = 28.2 (5)°] so that, overall, the mol­ecule has a flattened shape. The hy­droxy and amine groups are almost *syn* which enables the formation of inter­molecular hy­droxy-OH⋯N(thia­diazo­yl) and amine-H⋯O(sulfon­yl) hydrogen bonds leading to a supra­molecular chain aligned along the *a* axis.

## Related literature

For background to the use of amino alcohols in medicinal chemistry, see: Ferreira *et al.* (2009 ▶); de Oliveira *et al.* (2008 ▶); Brik & Wong (2003 ▶); Ghosh *et al.* (2001 ▶); Parikh *et al.* (2005 ▶); Andrews *et al.* (2006 ▶). For the anti-malarial activity of hy­droxy­ethyl­piperazines, see: Cunico, Gomes, Moreth *et al.* (2009 ▶). For the biological activity of hy­droxy­ethyl­sulfonamides, see: Cunico *et al.* (2008 ▶, 2011 ▶); Cunico, Gomes, Facchinetti *et al.* (2009 ▶). For related structures, see: Cunico, Gomes, Harrison *et al.* (2009 ▶); Gomes *et al.* (2011 ▶).
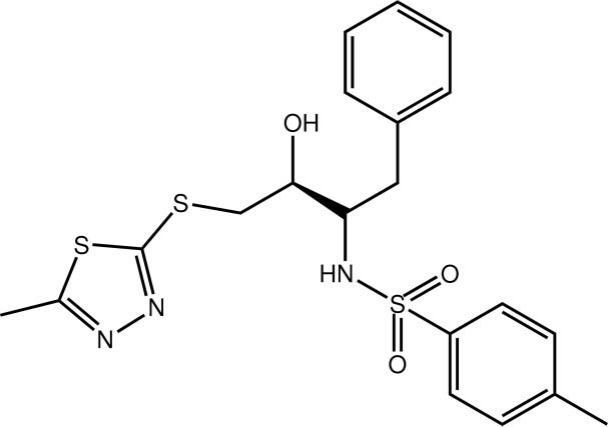


## Experimental

### 

#### Crystal data

C_20_H_23_N_3_O_3_S_3_
*M* *_r_* = 449.59
Orthorhombic, 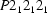

*a* = 5.0420 (2) Å
*b* = 18.4840 (8) Å
*c* = 22.9650 (8) Å
*V* = 2140.25 (15) Å^3^
*Z* = 4
Mo *K*α radiation
μ = 0.37 mm^−1^
*T* = 120 K
0.14 × 0.02 × 0.02 mm

#### Data collection

Bruker–Nonius Roper CCD camera on κ-goniostat diffractometer
Absorption correction: multi-scan (*SADABS*; Sheldrick, 2007 ▶) *T*_min_ = 0.438, *T*_max_ = 1.000
12594 measured reflections
2182 independent reflections
1538 reflections with *I* > 2σ(*I*)
*R*_int_ = 0.144

#### Refinement

*R*[*F* ^2^ > 2σ(*F* ^2^)] = 0.078
*wR*(*F* ^2^) = 0.206
*S* = 1.29
2182 reflections
270 parameters
2 restraints
H atoms treated by a mixture of independent and constrained refinement
Δρ_max_ = 0.48 e Å^−3^
Δρ_min_ = −0.47 e Å^−3^
Absolute structure: nd
Flack parameter: ?
Rogers parameter: ?

### 

Data collection: *COLLECT* (Hooft, 1998 ▶); cell refinement: *DENZO* (Otwinowski & Minor, 1997 ▶) and *COLLECT*; data reduction: *DENZO* and *COLLECT*; program(s) used to solve structure: *SHELXS97* (Sheldrick, 2008 ▶); program(s) used to refine structure: *SHELXL97* (Sheldrick, 2008 ▶); molecular graphics: *ORTEP-3* (Farrugia, 1997 ▶) and *DIAMOND* (Brandenburg, 2006 ▶); software used to prepare material for publication: *publCIF* (Westrip, 2010 ▶).

## Supplementary Material

Crystal structure: contains datablock(s) global, I. DOI: 10.1107/S1600536811033575/hb6366sup1.cif

Structure factors: contains datablock(s) I. DOI: 10.1107/S1600536811033575/hb6366Isup2.hkl

Supplementary material file. DOI: 10.1107/S1600536811033575/hb6366Isup3.cml

Additional supplementary materials:  crystallographic information; 3D view; checkCIF report

## Figures and Tables

**Table 1:** Hydrogen-bond geometry (Å, °)

*D*—H⋯*A*	*D*—H	H⋯*A*	*D*⋯*A*	*D*—H⋯*A*
O1—H1*O*⋯N1^i^	0.84 (7)	2.11 (8)	2.860 (11)	148 (8)
N3—H3*N*⋯O3^i^	0.88 (2)	2.05 (4)	2.902 (10)	163 (9)

Symmetry code: (i) 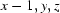.

**Fig. 1.**
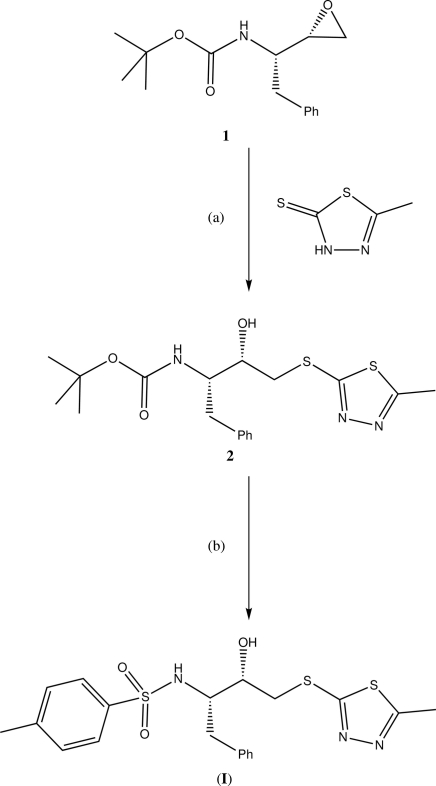
Synthesis. Reagents: (a) TFA/CH2Cl2 (1/3), RT, 6 h; (b) p-MeC6H4SO2Cl, Et3N, DMF, AcOEt, RT, 8 h.

**Fig. 2.**
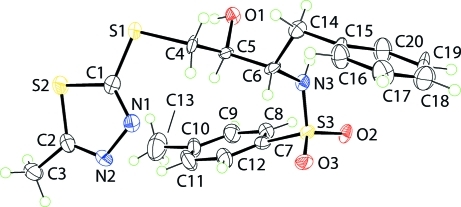
The molecular structure of (I) showing displacement ellipsoids at the 50% probability level.

**Fig. 3.**
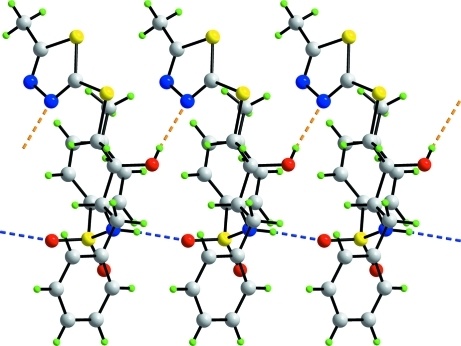
A view of the supramolecular chain along the a axis in (I). The O—H···N and N—H···O hydrogen bonding are shown as orange and blue dashed lines, respectively.

**Fig. 4.**
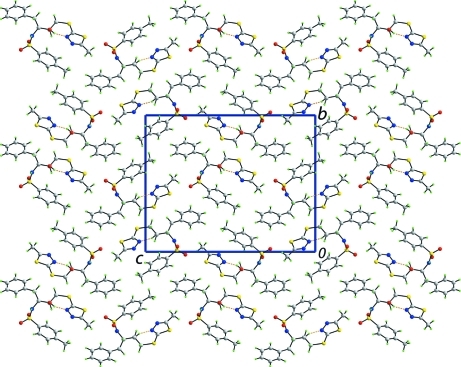
A view in projection down the a axis of the packing of supramolecular chains in (I). The The O—H···N hydrogen bonding is shown as orange dashed lines.

## supplementary crystallographic information

### Comment

Amino alcohols play versatile roles in medicinal chemistry (Ferreira *et al.*, 2009; de Oliveira *et al.*, 2008; Brik & Wong, 2003, Ghosh *et al.*, 2001; Parikh *et al.*, 2005; Andrews *et al.*, 2006). Some of us recently reported the anti-malarial activity of hydroxyethypiperazines against *Plasmodium falciparum* (Cunico, Gomes, Moreth *et al.*, 2009) and of hydroxyethylsulfonamide derivatives against *Plasmodium falciparum* (Cunico *et al.*, 2008; Cunico, Gomes, Facchinetti *et al.*, 2009), and mycobacterium tuberculosis H37Rv (Cunico *et al.*, 2011). In conjunction with these biological studies, crystal structure determinations of (2*R*,4*S*)-4-(arylmethyl)-1-(4-phenyl-3-amino-2-hydroxybutyl)-piperazine derivatives (Cunico, Gomes, Harrison *et al.*, 2009) and an example of a pyrimidyl derivative (Gomes *et al.*, 2011) have been carried out. In continuation of these structural studies, we now report the synthesis, Fig. 1, and structure of the title compound, (I).

In (I), Fig. 2, the thiadiazoyl and sulfonyl-benzene rings are orientated to the same side of the molecule but are not aligned in a parallel fashion as seen in the dihedral angle of 77.6 (5) ° formed between the rings. The benzyl-benzene is directed away from the rest of the twisted U-shaped molecule and forms a dihedral angle of 28.2 (5) ° with the sulfonyl-benzene ring. The key stereochemical feature of the molecule is the almost *syn* alignment of the hydroxyl and amine groups. This has an important consequence in the crystal packing.

As seen from Fig. 3, molecules assemble into a supramolecular chain *via* hydroxyl-OH···*N*(thiadiazoyl) and amine-*H*···*O*(sulfonyl) hydrogen bonds, Table 1. The chains are aligned along the *a* axis and assemble in the crystal structure without any specific interactions between them, Fig. 4.

### Experimental

Referring to Fig. 1, trifluoroacetic acid (1.5 ml, 20 mmol) was added to a solution of 2 (2 mmol), prepared from 1 and 5-methyl-1,3,4-thiadiazole-2(3*H*)-thione, in CH_2_Cl_2_ (6 ml). The mixture was stirred for 6 h, rotary evaporated to leave a residue, which was dissolved in EtOAc (20 ml), successively washed with 5% NaHCO_3_ aqueous solution, water and brine, and dried over MgSO_4_. The solvent was removed to afford the corresponding free amine, which was dissolved in EtOAc (10 mL) to which were added triethylamine (2.2 mmol) and *N*,*N*-dimethylformamide (0.2 mmol). The system was stirred for 30 minutes under nitrogen and *p*-toluenenesulfonyl chloride (2.0 mmol) was slowly added. The mixture was stirred for 8 h, successively washed with 5% HCl aqueous solution, water and brine, and dried over MgSO_4_. The solvent was removed in high vacuum and the title product 3 was obtained in 68% yield after recrystallization from hexane. The crystals used in the structure determination were grown from EtOH solution. *M*.pt: 412–414 K. EI—MS (m/*z*) (%): 472.1 (*M*++Na, 80%). ^1^H NMR [400.00 MHz, CDCl_3_] δ: 7.42 (d, 2H, J = 8.4 Hz, PhSO_2_); 7.11 (d, 2H, J = 8.0 Hz, PhSO_2_); 7.07–6.99 (m, 5H, Ph); 3.84–3.82 (m, 1H, H2); 3.58 (dd, 1H, ^1^J = 13.6 Hz, ^2^J = 4.4 Hz; H1b); 3.61–3.55 (m, 1H, H3); 3.22 (dd, 1H, ^1^J = 13.6 Hz, ^2^J = 8.4 Hz, H1a); 2.92 (dd, 1H, ^1^J = 14.0 Hz, ^2^J = 4.4 Hz, H4b); 2.72 (s, 3H, CH_3_(Het)); 2.59 (dd, ^1^H, J = 14.0 Hz, ^2^J = 8.4 Hz, H4a); 2.35 (s, 3H; CH_3_) p.p.m.. ^13^C NMR [100.0 MHz, CDCl_3_] δ: 168.6; 167.9; 144.1; 139.9; 139.2; 130.7; 129.4; 127.8; 127.3; 115.2; 73.4; 60.6; 39.4; 37.1; 21.6; 15.4 p.p.m. IR (cm^-1^; KBr pellets): ν_max_: 3273 (OH); 3023 (NH); 1330, 1161 (O═S═O); 686 (C—S).

### Refinement

The C-bound H atoms were geometrically placed (C–H = 0.95–1.00 Å) and refined as riding with *U*_iso_(H) = 1.2–1.5*U*_eq_(C). The O– and N-bound H atoms were located from a difference map and refined with the distance restraints O–H = 0.84 ± 0.01 and N–H = 0.88±0.01 Å, and with *U*_iso_(H) = *zU*_eq_(carrier atom); *z* = 1.5 for O and *z* = 1.2 for N. The refinement of the Flack absolute sturcture was ambiguous [refined value = 0.24 (19)] and 1482 Friedel pairs were averaged in the final refinement and the absolute configuration was assigned on the basis of the chirality of the *L*-serine starting material. The small (0.02 *x* 0.02 *x* 0.14 mm) needle was weakly diffracting but it was not deemed necessary to secure a data set with synchrotron radiation. The poor nature of the sample is also reflected in the relatively high values of *R*_int_ and in the residuals. However, the structure has been determined unambiguously.

### Crystal data

**Table:**

C_20_H_23_N_3_O_3_S_3_	*F*(000) = 944
*M**_r_* = 449.59	*D*_x_ = 1.395 Mg m^−^^3^
Orthorhombic, *P*2_1_2_1_2_1_	Mo *K*α radiation, λ = 0.71073 Å
Hall symbol: P 2ac 2ab	Cell parameters from 20956 reflections
*a* = 5.0420 (2) Å	θ = 2.9–27.5°
*b* = 18.4840 (8) Å	µ = 0.37 mm^−^^1^
*c* = 22.9650 (8) Å	*T* = 120 K
*V* = 2140.25 (15) Å^3^	Needle, colourless
*Z* = 4	0.14 × 0.02 × 0.02 mm

### Data collection

**Table:**

Bruker–Nonius Roper CCD camera on κ-goniostat diffractometer	2182 independent reflections
Radiation source: Bruker–Nonius FR591 rotating anode	1538 reflections with *I* > 2σ(*I*)
graphite	*R*_int_ = 0.144
Detector resolution: 9.091 pixels mm^-1^	θ_max_ = 25.0°, θ_min_ = 3.4°
φ & ω scans	*h* = −4→5
Absorption correction: multi-scan (*SADABS*; Sheldrick, 2007)	*k* = −20→21
*T*_min_ = 0.438, *T*_max_ = 1.000	*l* = −27→26
12594 measured reflections	

### Refinement

**Table:**

Refinement on *F*^2^	Secondary atom site location: difference Fourier map
Least-squares matrix: full	Hydrogen site location: inferred from neighbouring sites
*R*[*F*^2^ > 2σ(*F*^2^)] = 0.078	H atoms treated by a mixture of independent and constrained refinement
*wR*(*F*^2^) = 0.206	*w* = 1/[σ^2^(*F*_o_^2^) + (0.1*P*)^2^] where *P* = (*F*_o_^2^ + 2*F*_c_^2^)/3
*S* = 1.29	(Δ/σ)_max_ < 0.001
2182 reflections	Δρ_max_ = 0.48 e Å^−^^3^
270 parameters	Δρ_min_ = −0.47 e Å^−^^3^
2 restraints	Absolute structure: nd
Primary atom site location: structure-invariant direct methods	

### Special details

**Table:**

Geometry. All s.u.'s (except the s.u. in the dihedral angle between two l.s. planes) are estimated using the full covariance matrix. The cell s.u.'s are taken into account individually in the estimation of s.u.'s in distances, angles and torsion angles; correlations between s.u.'s in cell parameters are only used when they are defined by crystal symmetry. An approximate (isotropic) treatment of cell s.u.'s is used for estimating s.u.'s involving l.s. planes.
Refinement. Refinement of *F*^2^ against ALL reflections. The weighted *R*-factor *wR* and goodness of fit *S* are based on *F*^2^, conventional *R*-factors *R* are based on *F*, with *F* set to zero for negative *F*^2^. The threshold expression of *F*^2^ > 2σ(*F*^2^) is used only for calculating *R*-factors(gt) *etc*. and is not relevant to the choice of reflections for refinement. *R*-factors based on *F*^2^ are statistically about twice as large as those based on *F*, and *R*- factors based on ALL data will be even larger.

### Fractional atomic coordinates and isotropic or equivalent isotropic displacement parameters (Å^2^)

**Table:**

	*x*	*y*	*z*	*U*_iso_*/*U*_eq_	
S1	0.1474 (5)	0.17021 (13)	0.05038 (10)	0.0243 (6)	
S2	0.3692 (5)	0.07316 (15)	0.14153 (11)	0.0324 (7)	
S3	0.2827 (4)	0.01335 (13)	−0.18450 (11)	0.0222 (6)	
O1	−0.1857 (12)	0.1270 (3)	−0.0645 (3)	0.0230 (15)	
H1O	−0.23 (2)	0.097 (4)	−0.039 (3)	0.034*	
O2	0.1680 (13)	−0.0208 (3)	−0.2346 (3)	0.0246 (15)	
O3	0.5507 (12)	0.0390 (4)	−0.1872 (3)	0.0302 (17)	
N1	0.5551 (15)	0.0748 (5)	0.0380 (4)	0.027 (2)	
N2	0.7075 (16)	0.0234 (4)	0.0689 (4)	0.030 (2)	
N3	0.0990 (15)	0.0809 (4)	−0.1688 (3)	0.0205 (18)	
H3N	−0.074 (3)	0.078 (5)	−0.173 (4)	0.025*	
C1	0.3774 (19)	0.1027 (5)	0.0713 (4)	0.023 (2)	
C2	0.632 (2)	0.0174 (5)	0.1215 (4)	0.029 (2)	
C3	0.757 (2)	−0.0339 (6)	0.1642 (5)	0.036 (3)	
H3A	0.6187	−0.0634	0.1825	0.054*	
H3B	0.8512	−0.0063	0.1943	0.054*	
H3C	0.8827	−0.0655	0.1438	0.054*	
C4	0.215 (2)	0.1833 (5)	−0.0267 (4)	0.024 (2)	
H4A	0.1495	0.2316	−0.0384	0.028*	
H4B	0.4099	0.1829	−0.0326	0.028*	
C5	0.0909 (18)	0.1267 (5)	−0.0661 (4)	0.019 (2)	
H5	0.1553	0.0778	−0.0539	0.023*	
C6	0.1811 (18)	0.1406 (5)	−0.1295 (4)	0.020 (2)	
H6	0.3791	0.1436	−0.1299	0.024*	
C7	0.264 (2)	−0.0480 (5)	−0.1259 (4)	0.025 (2)	
C8	0.058 (2)	−0.0988 (6)	−0.1256 (5)	0.032 (3)	
H8	−0.0527	−0.1045	−0.1587	0.039*	
C9	0.020 (2)	−0.1406 (6)	−0.0763 (5)	0.038 (3)	
H9	−0.1215	−0.1746	−0.0750	0.046*	
C10	0.189 (3)	−0.1329 (6)	−0.0285 (5)	0.043 (3)	
C11	0.395 (3)	−0.0845 (6)	−0.0296 (5)	0.044 (3)	
H11	0.5110	−0.0797	0.0029	0.052*	
C12	0.431 (2)	−0.0421 (6)	−0.0799 (5)	0.033 (3)	
H12	0.5749	−0.0090	−0.0816	0.040*	
C13	0.135 (4)	−0.1739 (7)	0.0276 (6)	0.073 (5)	
H13A	0.2824	−0.1666	0.0546	0.110*	
H13B	0.1159	−0.2255	0.0190	0.110*	
H13C	−0.0296	−0.1559	0.0452	0.110*	
C14	0.071 (2)	0.2108 (5)	−0.1548 (5)	0.025 (2)	
H14A	−0.1230	0.2056	−0.1598	0.031*	
H14B	0.1017	0.2504	−0.1265	0.031*	
C15	0.190 (2)	0.2317 (5)	−0.2115 (4)	0.022 (2)	
C16	0.399 (2)	0.2813 (6)	−0.2132 (5)	0.034 (3)	
H16	0.4615	0.3017	−0.1777	0.041*	
C17	0.519 (2)	0.3018 (6)	−0.2654 (5)	0.038 (3)	
H17	0.6593	0.3359	−0.2655	0.046*	
C18	0.433 (2)	0.2729 (7)	−0.3151 (6)	0.045 (3)	
H18	0.5146	0.2859	−0.3509	0.054*	
C19	0.227 (3)	0.2243 (6)	−0.3145 (5)	0.047 (3)	
H19	0.1661	0.2042	−0.3501	0.056*	
C20	0.108 (3)	0.2043 (6)	−0.2634 (5)	0.039 (3)	
H20	−0.0348	0.1707	−0.2642	0.046*	

### Atomic displacement parameters (Å^2^)

**Table:**

	*U*^11^	*U*^22^	*U*^33^	*U*^12^	*U*^13^	*U*^23^
S1	0.0275 (13)	0.0300 (13)	0.0153 (13)	0.0022 (11)	−0.0008 (10)	−0.0003 (10)
S2	0.0342 (15)	0.0430 (16)	0.0201 (14)	0.0050 (13)	0.0017 (11)	0.0089 (11)
S3	0.0178 (12)	0.0274 (14)	0.0213 (13)	−0.0001 (10)	0.0010 (9)	−0.0063 (10)
O1	0.019 (3)	0.029 (4)	0.021 (4)	−0.005 (3)	0.002 (3)	0.006 (3)
O2	0.027 (4)	0.032 (4)	0.015 (3)	−0.010 (3)	0.000 (3)	−0.008 (3)
O3	0.017 (3)	0.045 (4)	0.029 (4)	0.002 (3)	0.001 (3)	−0.006 (3)
N1	0.014 (4)	0.044 (5)	0.022 (5)	−0.007 (4)	0.004 (3)	0.004 (4)
N2	0.027 (5)	0.035 (5)	0.028 (5)	0.009 (4)	−0.004 (4)	0.004 (4)
N3	0.017 (4)	0.026 (4)	0.018 (4)	−0.007 (3)	−0.003 (3)	−0.005 (3)
C1	0.018 (5)	0.027 (5)	0.024 (6)	−0.013 (4)	0.002 (4)	−0.003 (4)
C2	0.033 (6)	0.031 (6)	0.024 (6)	0.001 (5)	−0.008 (5)	0.003 (5)
C3	0.032 (6)	0.041 (6)	0.035 (7)	0.009 (5)	−0.002 (5)	0.007 (5)
C4	0.036 (6)	0.026 (5)	0.009 (5)	0.000 (5)	0.001 (4)	−0.003 (4)
C5	0.022 (5)	0.018 (5)	0.018 (5)	0.008 (4)	0.008 (4)	0.004 (4)
C6	0.018 (5)	0.021 (5)	0.022 (5)	0.002 (4)	0.002 (4)	−0.012 (4)
C7	0.028 (5)	0.020 (5)	0.026 (6)	0.009 (4)	0.002 (4)	−0.001 (4)
C8	0.029 (6)	0.043 (7)	0.026 (6)	0.006 (5)	−0.002 (5)	0.002 (5)
C9	0.051 (7)	0.030 (6)	0.034 (8)	0.006 (5)	−0.003 (5)	0.003 (5)
C10	0.069 (9)	0.039 (6)	0.021 (7)	0.024 (7)	−0.011 (6)	−0.004 (5)
C11	0.054 (8)	0.042 (7)	0.035 (7)	0.015 (6)	−0.021 (6)	−0.001 (5)
C12	0.038 (6)	0.032 (6)	0.029 (7)	−0.001 (5)	−0.012 (5)	−0.003 (5)
C13	0.123 (14)	0.057 (9)	0.040 (9)	0.016 (10)	0.001 (9)	0.005 (7)
C14	0.030 (6)	0.019 (5)	0.027 (6)	−0.002 (4)	0.004 (4)	−0.002 (4)
C15	0.030 (6)	0.025 (5)	0.011 (5)	0.008 (4)	−0.001 (4)	−0.004 (4)
C16	0.033 (6)	0.042 (7)	0.027 (6)	−0.010 (5)	−0.003 (5)	0.006 (5)
C17	0.040 (7)	0.036 (7)	0.038 (8)	−0.011 (5)	−0.003 (5)	0.009 (6)
C18	0.048 (7)	0.050 (8)	0.036 (8)	0.003 (6)	0.012 (6)	0.012 (6)
C19	0.089 (10)	0.041 (7)	0.010 (6)	0.002 (7)	0.007 (6)	0.001 (5)
C20	0.048 (7)	0.039 (6)	0.030 (7)	−0.009 (5)	0.001 (6)	0.009 (5)

### Geometric parameters (Å, °)

**Table:**

S1—C1	1.770 (10)	C7—C8	1.397 (14)
S1—C4	1.818 (9)	C8—C9	1.384 (16)
S2—C1	1.704 (10)	C8—H8	0.9500
S2—C2	1.742 (11)	C9—C10	1.397 (16)
S3—O3	1.433 (7)	C9—H9	0.9500
S3—O2	1.435 (6)	C10—C11	1.374 (18)
S3—N3	1.596 (8)	C10—C13	1.518 (18)
S3—C7	1.763 (10)	C11—C12	1.407 (16)
O1—C5	1.395 (11)	C11—H11	0.9500
O1—H1O	0.838 (11)	C12—H12	0.9500
N1—C1	1.285 (12)	C13—H13A	0.9800
N1—N2	1.414 (11)	C13—H13B	0.9800
N2—C2	1.271 (13)	C13—H13C	0.9800
N3—C6	1.485 (11)	C14—C15	1.484 (14)
N3—H3N	0.878 (11)	C14—H14A	0.9900
C2—C3	1.504 (14)	C14—H14B	0.9900
C3—H3A	0.9800	C15—C20	1.361 (15)
C3—H3B	0.9800	C15—C16	1.398 (14)
C3—H3C	0.9800	C16—C17	1.395 (16)
C4—C5	1.520 (13)	C16—H16	0.9500
C4—H4A	0.9900	C17—C18	1.332 (17)
C4—H4B	0.9900	C17—H17	0.9500
C5—C6	1.546 (13)	C18—C19	1.374 (17)
C5—H5	1.0000	C18—H18	0.9500
C6—C14	1.526 (13)	C19—C20	1.369 (16)
C6—H6	1.0000	C19—H19	0.9500
C7—C12	1.357 (15)	C20—H20	0.9500
			
C1—S1—C4	103.5 (5)	C8—C7—S3	118.4 (8)
C1—S2—C2	85.4 (5)	C9—C8—C7	118.9 (11)
O3—S3—O2	119.4 (4)	C9—C8—H8	120.6
O3—S3—N3	107.4 (4)	C7—C8—H8	120.6
O2—S3—N3	107.0 (4)	C8—C9—C10	120.0 (12)
O3—S3—C7	107.3 (5)	C8—C9—H9	120.0
O2—S3—C7	107.9 (4)	C10—C9—H9	120.0
N3—S3—C7	107.4 (4)	C11—C10—C9	120.9 (11)
C5—O1—H1O	105 (8)	C11—C10—C13	118.5 (12)
C1—N1—N2	110.5 (8)	C9—C10—C13	120.4 (14)
C2—N2—N1	111.9 (8)	C10—C11—C12	118.3 (10)
C6—N3—S3	123.9 (6)	C10—C11—H11	120.8
C6—N3—H3N	112 (6)	C12—C11—H11	120.8
S3—N3—H3N	121 (6)	C7—C12—C11	121.0 (10)
N1—C1—S2	116.9 (8)	C7—C12—H12	119.5
N1—C1—S1	125.3 (8)	C11—C12—H12	119.5
S2—C1—S1	117.8 (6)	C10—C13—H13A	109.5
N2—C2—C3	123.4 (10)	C10—C13—H13B	109.5
N2—C2—S2	115.3 (7)	H13A—C13—H13B	109.5
C3—C2—S2	121.3 (8)	C10—C13—H13C	109.5
C2—C3—H3A	109.5	H13A—C13—H13C	109.5
C2—C3—H3B	109.5	H13B—C13—H13C	109.5
H3A—C3—H3B	109.5	C15—C14—C6	114.1 (8)
C2—C3—H3C	109.5	C15—C14—H14A	108.7
H3A—C3—H3C	109.5	C6—C14—H14A	108.7
H3B—C3—H3C	109.5	C15—C14—H14B	108.7
C5—C4—S1	114.2 (7)	C6—C14—H14B	108.7
C5—C4—H4A	108.7	H14A—C14—H14B	107.6
S1—C4—H4A	108.7	C20—C15—C16	116.7 (9)
C5—C4—H4B	108.7	C20—C15—C14	123.3 (9)
S1—C4—H4B	108.7	C16—C15—C14	120.0 (9)
H4A—C4—H4B	107.6	C17—C16—C15	121.9 (10)
O1—C5—C4	113.2 (8)	C17—C16—H16	119.1
O1—C5—C6	108.6 (8)	C15—C16—H16	119.1
C4—C5—C6	109.0 (7)	C18—C17—C16	119.3 (11)
O1—C5—H5	108.7	C18—C17—H17	120.4
C4—C5—H5	108.7	C16—C17—H17	120.4
C6—C5—H5	108.7	C17—C18—C19	119.8 (12)
N3—C6—C14	107.5 (8)	C17—C18—H18	120.1
N3—C6—C5	111.4 (8)	C19—C18—H18	120.1
C14—C6—C5	113.1 (7)	C20—C19—C18	121.2 (12)
N3—C6—H6	108.2	C20—C19—H19	119.4
C14—C6—H6	108.2	C18—C19—H19	119.4
C5—C6—H6	108.2	C15—C20—C19	121.1 (11)
C12—C7—C8	120.7 (10)	C15—C20—H20	119.4
C12—C7—S3	120.7 (8)	C19—C20—H20	119.4
			
C1—N1—N2—C2	−0.1 (12)	O3—S3—C7—C8	−158.1 (8)
O3—S3—N3—C6	−35.0 (9)	O2—S3—C7—C8	−28.3 (9)
O2—S3—N3—C6	−164.3 (7)	N3—S3—C7—C8	86.7 (8)
C7—S3—N3—C6	80.1 (8)	C12—C7—C8—C9	3.3 (15)
N2—N1—C1—S2	0.5 (10)	S3—C7—C8—C9	−171.4 (8)
N2—N1—C1—S1	179.0 (6)	C7—C8—C9—C10	−1.5 (16)
C2—S2—C1—N1	−0.6 (8)	C8—C9—C10—C11	−0.5 (18)
C2—S2—C1—S1	−179.2 (6)	C8—C9—C10—C13	174.9 (11)
C4—S1—C1—N1	3.4 (9)	C9—C10—C11—C12	0.7 (17)
C4—S1—C1—S2	−178.2 (5)	C13—C10—C11—C12	−174.8 (11)
N1—N2—C2—C3	179.3 (9)	C8—C7—C12—C11	−3.1 (16)
N1—N2—C2—S2	−0.3 (11)	S3—C7—C12—C11	171.4 (8)
C1—S2—C2—N2	0.5 (8)	C10—C11—C12—C7	1.1 (17)
C1—S2—C2—C3	−179.1 (9)	N3—C6—C14—C15	−66.3 (10)
C1—S1—C4—C5	80.3 (8)	C5—C6—C14—C15	170.2 (8)
S1—C4—C5—O1	63.4 (9)	C6—C14—C15—C20	82.7 (12)
S1—C4—C5—C6	−175.7 (6)	C6—C14—C15—C16	−96.6 (11)
S3—N3—C6—C14	141.8 (7)	C20—C15—C16—C17	−0.1 (16)
S3—N3—C6—C5	−93.7 (9)	C14—C15—C16—C17	179.2 (10)
O1—C5—C6—N3	−64.8 (9)	C15—C16—C17—C18	−0.6 (17)
C4—C5—C6—N3	171.5 (7)	C16—C17—C18—C19	0.9 (18)
O1—C5—C6—C14	56.5 (10)	C17—C18—C19—C20	−0.5 (19)
C4—C5—C6—C14	−67.3 (10)	C16—C15—C20—C19	0.5 (16)
O3—S3—C7—C12	27.3 (10)	C14—C15—C20—C19	−178.8 (11)
O2—S3—C7—C12	157.1 (8)	C18—C19—C20—C15	−0.2 (19)
N3—S3—C7—C12	−87.9 (9)		

### Hydrogen-bond geometry (Å, °)

**Table:**

*D*—H···*A*	*D*—H	H···*A*	*D*···*A*	*D*—H···*A*
O1—H1O···N1^i^	0.84 (7)	2.11 (8)	2.860 (11)	148 (8)
N3—H3N···O3^i^	0.88 (2)	2.05 (4)	2.902 (10)	163 (9)

Symmetry codes: (i) *x*−1, *y*, *z*.
